# The Role of Indoor Microbiome and Metabolites in Shaping Children’s Nasal and Oral Microbiota: A Pilot Multi-Omic Analysis

**DOI:** 10.3390/metabo13101040

**Published:** 2023-09-27

**Authors:** Mei Zhang, Hao Tang, Yiwen Yuan, Zheyuan Ou, Zhuoru Chen, Yanyi Xu, Xi Fu, Zhuohui Zhao, Yu Sun

**Affiliations:** 1 Guangdong Provincial Key Laboratory of Protein Function and Regulation in Agricultural Organisms, College of Life Sciences, South China Agricultural University, Guangzhou 510642, China; zmbliss@stu.scau.edu.cn (M.Z.); yyw_324124@stu.scau.edu.cn (Y.Y.); the_o.scau.edu.cn@stu.scau.edu.cn (Z.O.); 2 School of Public Health, Fudan University, Shanghai 200032, China; 19211020050@fudan.edu.cn (H.T.); yanyi_xu@fudan.edu.cn (Y.X.); 3 Children’s Hospital of Fudan University, Shanghai 201102, China; 19211020086@fudan.edu.cn; 4 Guangdong Provincial Engineering Research Center of Public Health Detection and Assessment, School of Public Health, Guangdong Pharmaceutical University, Guangzhou 510006, China; fuxi@gdpu.edu.cn; 5 Key Laboratory of Public Health Safety of the Ministry of Education, NHC Key Laboratory of Health Technology Assessment (Fudan University), Shanghai Typhoon Institute/CMA, Shanghai Key Laboratory of Meteorology and Health, Shanghai 200030, China

**Keywords:** flavonoids, mycotoxin, ETS, protective/risk microorganisms, microbial transfer, multi-omics

## Abstract

Maintaining a diverse and well-balanced nasal and oral microbiota is vital for human health. However, the impact of indoor microbiome and metabolites on nasal and oral microbiota remains largely unknown. Fifty-six children in Shanghai were surveyed to complete a questionnaire about their personal and environmental characteristics. The indoor microbiome and metabolites from vacuumed indoor dust were profiled via shotgun metagenomics and untargeted liquid chromatography-mass spectrometry (LC–MS). The nasal and oral microbiota in children was characterized using full-length 16S rRNA sequencing from PacBio. Associations between personal/environmental characteristics and the nasal/oral microbiota were calculated using PERMANOVA and regression analyses. We identified 6247, 431, and 342 microbial species in the indoor dust, nasal, and oral cavities, respectively. The overall nasal and oral microbial composition showed significant associations with environmental tobacco smoke (ETS) exposure during pregnancy and early childhood (*p* = 0.005 and 0.03, respectively), and the abundance of total indoor flavonoids and two mycotoxins (deoxynivalenol and nivalenol) (*p* = 0.01, 0.02, and 0.03, respectively). Notably, the abundance of several flavonoids, such as baicalein, eupatilin, isoliquiritigenin, tangeritin, and hesperidin, showed positive correlations with alpha diversity and the abundance of protective microbial taxa in nasal and oral cavities (*p* < 0.02), suggesting their potential beneficial roles in promoting nasal/oral health. Conversely, high carbohydrate/fat food intake and ETS exposure diminished protective microorganisms while augmenting risky microorganisms in the nasal/oral cavities. Further, potential microbial transfer was observed from the indoor environment to the childhood oral cavity (*Moraxella catarrhalis*, *Streptococcus mitis*, and *Streptococcus salivarius*), which could potentially increase virulence factors related to adherence and immune modulation and vancomycin resistance genes in children. This is the first study to reveal the association between the indoor microbiome/metabolites and nasal/oral microbiota using multi-omic approaches. These findings reveal potential protective and risk factors related to the indoor microbial environment.

## 1. Introduction

The nasal cavity, a critical respiratory organ in humans and animals, facilitates essential respiratory functions such as air exchange, filtering, humidifying, and warming. It habitats a diverse microbial community encompassing numerous bacterial phyla, including Firmicutes, Actinobacteria, Bacteroidetes, Proteobacteria, and Fusobacteria [1,2]. The stability of this nasal microbiota plays a fundamental role in maintaining respiratory health, and disturbances or dysbiosis of this microbial community have been implicated in a variety of acute and chronic respiratory diseases, such as rhinosinusitis [3,4] and asthma [5]. For instance, an increased abundance of the bacterial genus *Moraxella* has been associated with exacerbations of asthma and chronic obstructive pulmonary disease [6]. Conversely, certain microbes like *Corynebacterium* and *Dolosigranulum* are thought to have protective roles against such respiratory diseases [7,8]. Furthermore, high microbial alpha diversity/richness within the nasal microbiome has been suggested to confer protective effects against nasal diseases [9].

The oral cavity, acting as the secondary external opening for the respiratory tract and the initial site of the digestive tract, is similarly colonized by a complex microbiota. Unlike the nasal cavity, which acts as a filter and often captures environmental species that do not establish long-term colonization, the oral cavity has a higher abundance of truly residential microorganisms. There are roughly eight hundred microbial species that have been characterized in the oral cavity [10]. This oral microbiota exhibits substantial spatial compositional variation across different oral niches [11]. Dysbiosis within this community has been correlated with numerous oral diseases, including gingivitis, periodontitis, and dental caries [12,13,14], significantly impacting medical and economic burdens and lowering the overall quality of life. Importantly, alterations in the oral microbiome have also been linked to respiratory diseases, such as pneumonia and chronic obstructive pulmonary disease (COPD), owing to the aspiration of oral pathogens into the lower respiratory tract [15]. A decreased oral microbial alpha diversity has been associated with severe respiratory conditions, such as lung cancer [16]. Thus, maintaining a healthy nasal and oral microbiome is of paramount importance for human health.

Understanding the factors influencing the nasal and oral microbiota is vital to improving human health. Several personal and environmental characteristics, including host genetic background, age and gender, air pollution, volatile environmental chemicals surrounding greenness, antibiotic usage, environmental tobacco smoke (ETS), and geographic regions [17,18,19,20,21,22,23,24,25], have been reported to impact the nasal and oral microbial composition. However, the effects of indoor microbiome/metabolites on the diversity and abundance of health-related nasal/oral microbiota remain largely unexplored. The indoor microbiome and microbial metabolites have been linked to several acute and chronic respiratory diseases, including respiratory infections, asthma, rhinitis, and sick-building syndrome [26,27,28,29,30,31,32], underscoring the necessity for in-depth research to elucidate potential interactions and connections between the indoor microbiome/metabolites and nasal/oral microbiota.

In this study, we conducted a comprehensive assessment of the association between personal and environmental characteristics and children’s nasal and oral microbiomes. Specifically, we explored the impact of indoor microbial exposure (microbiome and metabolites), characteristics of the living environment, air pollution, and personal characteristics (including personal, family, and food intake information) on the diversity of nasal/oral microbiome and health-related microorganisms. Additionally, we investigated the potential transference of microbial taxa and functional genes (including virulence genes and antimicrobial resistance genes) from the indoor environment to the nasal/oral cavities. To our knowledge, this is the first study to report the association between indoor microbiome/metabolome exposure and nasal/oral microbiota, thereby offering novel insights into human health.

## 2. Materials and Methods

### 2.1. Data and Sample Collection

From December 2019 to April 2020, we recruited 56 healthy children, mainly aged between 4 and 6 years, who had resided in Shanghai, China, for at least one year. Participants were identified and recruited through local schools and community centers. A screening questionnaire was administered to parents to ensure that the children met the eligibility criteria, including being in good health and having lived in Shanghai for the requisite period. To determine the health status of the participants, children were required to have no history of major illnesses, chronic conditions, or antibiotic usage in the past year. Although our sample size is limited, the demographic characteristics of the participants closely match those of the broader population of 4–6-year-old children living in Shanghai, thereby enhancing the generalizability of our findings. A self-reported questionnaire was used to gather information on personal details, family and parental background, environmental factors, and frequency of food intake. The questionnaire was adapted from the International Study of Asthma and Allergies in Children [33]. Additionally, the annual outdoor air pollutants were determined using the inverse distance weighted (IDW) method, which utilized data from local air monitoring stations [34,35]. Prior to data collection, the purpose of the study was explained to the children and their guardians, and informed consent was obtained from all participants. The study design and protocol were approved by the ethical committee of the School of Public Health at Fudan University (IRB#2019-09-0778).

To collect indoor dust samples, we employed a standardized vacuuming procedure across all participating households. A sterile sampler with a 6 µm filter pore was equipped with a vacuum cleaner with 20 kPa pressure. For each home, the sampler was run for four minutes on the living room floor and the child’s bed to collect dust. In instances where siblings shared a room, only the proband (child participating in the study) bed was sampled. The vacuum cleaner was placed in direct contact with the surfaces, ensuring that dust particles were effectively captured. The four-minute sampling length was determined based on previous research indicating that this duration is sufficient for obtaining a representative sample of indoor dust [30,36]. The sampling length was strictly adhered to for all households to maintain consistency in the sampling procedure. The fine dust was obtained by sieving the vacuumed dust through a 0.3 mm mesh screen and stored at −80 °C in a freezer. Nasal samples were collected from both nasal cavities using an Isohelix nasal cotton swab (rotating 15 times), which was then stored in a microtube at −80 °C until DNA extraction. Oral samples were collected using a saliva tube (SARSTEDT AG & Co., KG Sarstedtstraße, Nümbrecht, Germany). The children rinsed their mouths with water three to five times before chewing on a sponge for 60 s. The chewed sponge was then centrifuged at 1000 r/min for 2 min at 4 °C, and the saliva samples were stored at −80 °C until DNA extraction and sequencing. The collection of indoor vacuum dust, nasal swabs, and oral samples was conducted within a single day.

### 2.2. DNA Extraction, High-Throughput Sequencing and Microbiome Analysis

We utilized the Dneasy PowerSoil Kit from QIAGEN, Hilden, Germany, to extract a fifty-milligram sample of indoor dust for whole-genome shotgun metagenomic sequencing. The sequencing was performed by Personal Biotechnology Co., Ltd., a sequencing service provider based in Shanghai, China. The TruSeq DNA High-throughput Library Preparation Kit was used to construct a sequencing library with 2 × 150 bp paired-end reads and an insert size of 400 bp. A dual-indexed code was added to the constructed read for multiplexing. The prepared library was sequenced on the Illumina HiSeq X-ten platform (Illumina, San Diego, CA, USA). We deposited the raw sequencing data in the Genome Sequence Archive [37] under the accession number PRJCA008482. Cutadapt (v1.2.1), KneadData (v.0.9.0), and BMTagger (v3.1.01) were used to remove low-quality, chimeric, and human-derived reads. The clean reads were then assembled using MEGAHIT (v1.0.5) [38]. MetaGeneMark (v3.25) was used to predict coding sequences with a length > 300 bp [39]. To annotate the indoor microbial taxonomy, we compared the assembled sequences to the NCBI-NT database using BLASTN, setting an e-value threshold of less than 0.001. We then processed the resulting BLAST hits using MEGAN (v6.0), employing the Lowest Common Ancestor (LCA) assignment algorithm [40]. The virulence factors (VFs) and antimicrobial resistance genes (ARGs) were annotated by searching the clean reads in functional gene databases VFDB (Virulence Factor Database) [41] and CARD (Comprehensive Antibiotic Resistance Database) [42]. We measured the abundance of VFs and ARGs as reads per kilobase per million mapped reads (RPKM). To ensure the accuracy of our findings, we established a minimum threshold for sequence identity (70% in nucleotide sequences) and alignment length (50%). This helped us confirm significant matches and eliminate false positives or matches resulting from random sequence similarity.

Full-length bacterial 16S rRNA amplicon sequencing was conducted for nasal and oral saliva samples. DNA was extracted with a QIAamp DNA Microbiome Kit (QIAGEN, Hilden, Germany). Poly adenine and indexed read adapters were added at both ends, and the library was purified using Ampure PB beads. The SMRTbellTM Template Prep Kit 1.0 (PacBio, Menlo Park, CA, USA) was used to construct the sequencing library and sequenced on the PacBio Sequel II platform using the circular consensus sequencing (CCS) technique [43]. The following analysis was mainly conducted by the QIIME2 platform [44]. Raw sequencing reads were denoised by DADA2 [45]. The OTU taxonomy assignment was conducted based on searching a downloaded human oral microbiome database (HOMD database, version 15.23) [10]. Finally, we calculated the nasal and oral microbial compositional variation (beta diversity) using Bray–Curtis distance metrics.

### 2.3. Indoor Dust LC/MS for Metabolomics Profiling

The fine dust was also subjected to metabolic profiling using LC–MS on a Vanquish UHPLC system coupled with a QE-HF-X Orbitrap mass spectrometer from Thermo Fisher Scientific (Waltham, MA, USA). This process involved adding 2-chloropheylalanine to the fine dust and rotating the mixture for 30 s at −20 °C. We then filtered the supernatant through a 0.22 μm pore-sized membrane. Chromatographic separation was conducted by a column with a size of 150 × 2.1 mm × 1.8 μm (ACQUITY UPLC^®^ HSS T3) at 40 °C, and analytes were detected in water and acetonitrile at a flow rate of 0.25 mL/min. The mass spectrometer was set to scan over the mass range of 81–1000 *m*/*z* at a resolution of 60,000. Unnecessary information was removed from the MS/MS spectra by dynamic exclusion. The measurements were recorded as relative abundances presented as intensity values. Lastly, we aligned the analytes to several databases, including the Human Metabolome Database, METLIN, MoNA, mzCloud, and MassBank.

### 2.4. Environmental Characteristics and Association Analysis

We analyzed over 40 environmental characteristics in the association analysis, which can be broadly categorized into six major categories:

(1) Personal and family data, including, but not limited to, gender, age to start kindergarten, breastfeeding duration, age of child, premature delivery, type of delivery (premature or cesarean section), presence of siblings, and parental income and education level. These variables were self-reported and treated as categorical (e.g., gender) or continuous (e.g., age, income).
(2) Living environment characteristics, including room cleaning frequency, the age of the residential building, number of cohabitants, proximity to heavy traffic, rivers, parks, or gardens, presence of indoor pets, presence of indoor plants, maternal and child exposure to smoking, visible mold/dampness, and family history of *Helicobacter pylori* infection. Data were self-reported and categorized based on pre-defined criteria.
(3) Food intake frequency, including intake of meat, milk, eggs, seafood, fruits, salad, cooked vegetables, juice, soft drinks, fries, rice/pasta/bread. The data were self-reported and treated as ordinal variables based on intake frequency.
(4) The concentration of annual average outdoor air pollutants, including SO_2_, NO_2_, CO, O_3_, PM_10_, and PM_2.5_. The daily average values of atmospheric pollutants were collected from the environmental monitoring station closest to the children’s residences over a period of one year preceding the collection of biological samples.
(5) Indoor microbial exposure, including indoor microbial abundance and diversity, including the alpha diversity of the indoor microbiome (Shannon index, Chao1, observed a number of species), microbial virulence factors, antimicrobial resistance genes, and NIAID-defined pathogen species https://www.niaid.nih.gov/research/emerging-infectious-diseases-pathogens (accessed on 26 September 2023). These data were calculated from indoor shotgun metagenomics sequencing.
(6) Indoor metabolites come from four classes, including keto acids, indoles, flavonoids, and mycotoxins. These data were calculated from indoor metabolomic profiling. To address the compositional nature of the microbiome and metabolome data, we employed a centered log-ratio (CLR) transformation prior to conducting regression analyses.

In the statistical analyses, each of the independent variables was tested individually against the dependent variables. Specifically, we employed linear regression analyses using SPSS (Statistics 21) and StataIC15, adjusting for the children’s age and gender. The command line used for these analyses was “regress dependent–variable on independent–variable, age, and gender”. Independent variables were drawn from six categories of personal and environmental characteristics, and dependent variables included the diversity and relative abundance of risk-associated and protective microbial taxa within the nasal and oral cavities. In addition to the linear regression analyses, a permutational MANOVA (PERMANOVA) was conducted using the Adonis function in the R package with 10,000 permutations.

### 2.5. Potential Microbial Transfer between Indoor Environment to Nasal/Oral Cavities

We defined the potential transfer species from the indoor environment to the nasal/oral cavities as follows: (1) microbial indoor abundance > 0.2%; (2) microbial indoor abundance > microbial abundance in the nasal/oral cavities. Species with an indoor abundance lower than that in the nasal/oral cavities may indicate a reverse transfer from these cavities to the indoor environment; (3) a positive Spearman’s rank correlation (rho) with a *p*-value < 0.10. The VFs and ARGs of the three potential transfer species (*M. catarrhalis*, *S. mitis*, and *S. salivarius*) were characterized by searching against the CARD and VFDB databases.

## 3. Results

### 3.1. Personal Information, Environmental Characteristics and Dietary Frequency

In this study, a total of 56 children were randomly selected from twelve distinct districts in Shanghai, China. An assortment of personal, environmental (including indoor characteristics and air pollutants), and dietary characteristics were collected from the participating children, as detailed in Table 1. Among the cohort, approximately 59% were girls. Birth histories revealed that 12% of these children were born prematurely, while 45% were delivered via cesarean section. For environmental tobacco smoke (ETS) exposure, we found that 18% of the children were exposed during pregnancy, 14% during early childhood, and 18% in the preceding ten months.

### 3.2. Indoor Microbiome, VFs, ARGs, and Metabolites

**Figure 1:**
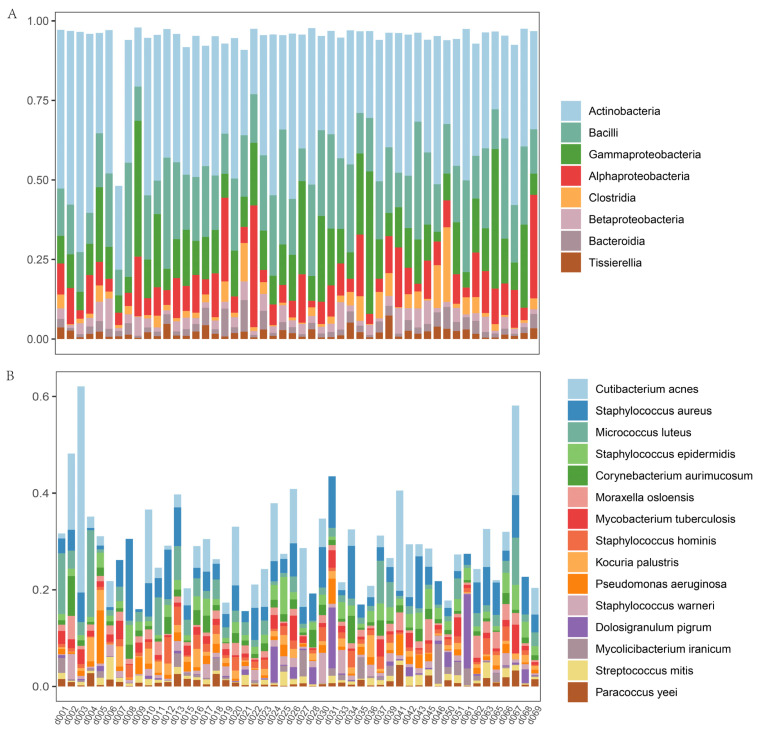
(**A**) The top 8 microbial classes and (**B**) the top 15 microbial species of the indoor environment. The *x*-axis lists individual sample identifiers, while the *y*-axis denotes the relative abundance of microbial taxa in the respective samples.

We characterized 6247, 431, and 342 microbial species from the indoor dust, nasal, and oral cavities, respectively. Actinobacteria, Bacilli, and Gammaproteobacteria were identified as the most prevalent indoor microbial classes, followed by Alphaproteobacteria, Clostridia, Betaproteobacteria, Bacteroidia, and Tissierella (Figure 1A). In terms of species, *Cutibacterium acnes* (5.26%), *Staphylococcus aureus* (4.72%), *Micrococcus luteus* (2.91%), and *Staphylococcus epidermidis* (2.16%) were found to be the most abundant (Figure 1B). Compared to bacteria, fungi were less abundant in the indoor environment. The most prevalent fungal species included *Malassezia restricta* (0.46%), *Malassezia globosa* (0.22%), and *Alternaria alternata* (0.21%). We also detected potential pathogens in the indoor dust, including *Clostridium perfringens* (0.01%), *Salmonella enterica* (0.50%), *Listeria monocytogenes* (0.01%), *Toxoplasma gondii* (0.01%), and *Mycobacterium tuberculosis* (1.47%) (Table 1).

The top VF categories identified were nutritional and metabolic factors (average RPKM 998.3, mainly including pyoverdine, FbpABC, and Acinetobactin), immune modulation (RPKM 641.7, including LOS, LPS, and Capsule I), and adherence (RPKM 433.4, including Type IV pili, Type 3 fimbriae, and Type 1 fimbriae). The most dominant ARG category was antibiotic efflux (RPKM 2661.9, including *macB*, *tetA*, *evgS*, *ranA*, *bcrA*, and *novA*), followed by antibiotic inactivation (RPKM 345.9, including *nmcR*) and antibiotic target alteration (RPKM 851.0, including *basS*).

A variety of indoor metabolites were detected, such as indole and keto acid derivatives, flavonoids, and mycotoxins. Table 1 shows the relative abundance (represented as the intensity value) of highly abundant representative indole and keto acid derivatives, flavonoids, and mycotoxins in the indoor dust.

### 3.3. Nasal and Oral Microbial Composition

**Figure 2:**
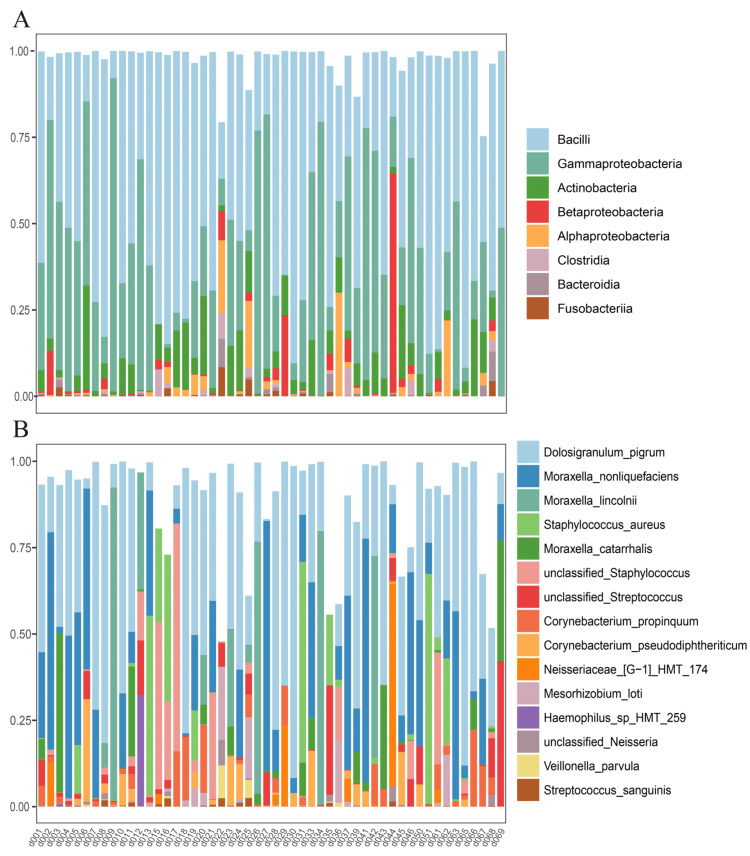
(**A**) The top 8 microbial classes and (**B**) the top 15 microbial species of the nasal microbiota. The *x*-axis lists individual sample identifiers, while the *y*-axis denotes the relative abundance of microbial taxa in the respective samples.

**Figure 3:**
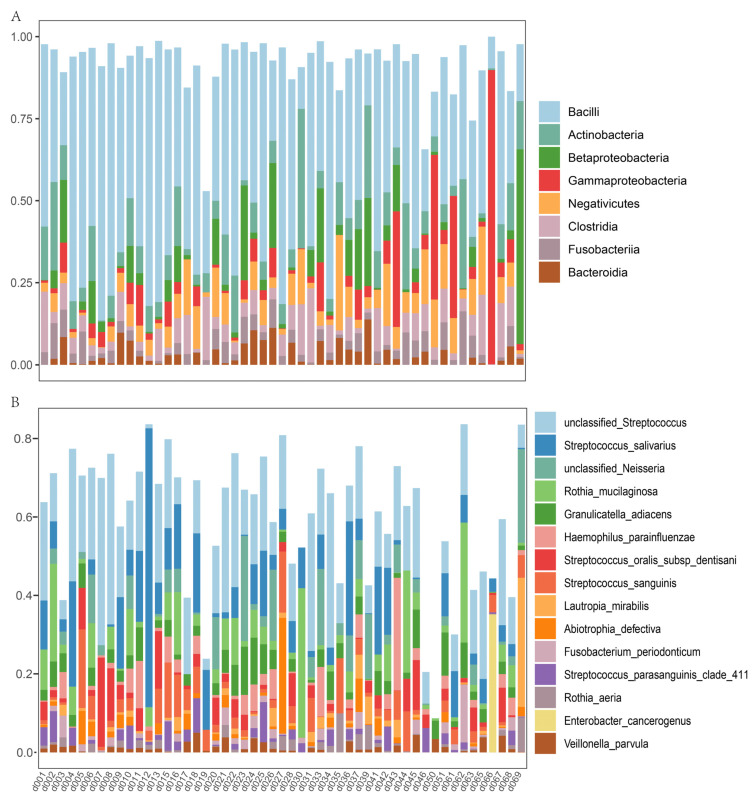
(**A**) The top 8 microbial classes and (**B**) the top 15 microbial species of the oral microbiota. The *x*-axis lists individual sample identifiers, while the *y*-axis denotes the relative abundance of microbial taxa in the respective samples.

In the nasal microbiome, Bacilli, Gammaproteobacteria, Actinobacteria, and Betaproteobacteria were highly abundant microbial classes (Figure 2A). The top nasal microbial species included *Dolosigranulum pigrum*, *Moraxella nonliquefaciens*, *Moraxella lincolnii*, *Staphylococcus aureus*, *Moraxella catarrhalis*, an unclassified *Staphylococcus*, and an unclassified *Streptococcus* (Figure 2B). In oral saliva, the top microbial classes were Bacilli, Actinobacteria, Betaproteobacteria, and Gammaproteobacteria. The top oral species included an unclassified *Streptococcus*, *Streptococcus salivarius*, an unclassified *Neisseria*, *Rothia mucilaginosa*, *Granulicatella adiacens*, *Haemophilus parainfluenzae*, *Streptococcus oralis*, *Streptococcus sanguinis*, and *Lautropia mirabilis* (Figure 3B).

### 3.4. Impact of Environmental Variables on Overall Nasal and Oral Microbial Composition

We used PERMANOVA to calculate the impact of environmental characteristics on the beta diversity (composition) of the nasal and oral microbiota (Table 1). To control for false positive results, only associations with a *p*-value < 0.02 were considered significant and presented in bold fonts. Age (*p* = 0.008, R^2^ = 4.2) and education level of the father (*p* = 0.04, R^2^ = 2.8) were significantly associated with the nasal microbial composition. Exposure to ETS during the mother’s pregnancy and early childhood was associated with nasal and oral microbial composition (*p* = 0.005 and 0.03, R^2^ = 4.63 and 1.74). Food intake, outdoor air pollution, the abundance of indoor pathogens, and the indoor total abundance of VFs and ARGs were not associated with the beta diversity of the nasal and oral microbiota. The abundance of total flavonoids was associated with the nasal microbial composition (*p* = 0.01, R^2^ = 3.90). Indole derivatives 3-methylindole (*p* = 0.01, R^2^ = 3.57), mycotoxin deoxynivalenol (*p* = 0.017, R^2^ = 3.26), and nivalenol (*p* = 0.028, R^2^ = 2.77) were also associated with nasal and oral microbial composition.

### 3.5. Impact of Environmental Variables on Alpha Diversity and the Abundance of Risky/Protective Nasal and Oral Microorganisms

Regression analyses were performed to evaluate the relationship between environmental variables and nasal alpha diversity, as characterized by the Shannon index and the observed species (Table 2). The presence of siblings (*p* = 0.008, β = 0.69) and exposure to an indoor flavonoid baicalein (*p* = 0.004, β = 0.72) were positively associated with the Shannon index. The presence of siblings (*p* = 0.01, β = 29.9) and eupatilin (*p* = 0.0037, β = 0.68; a flavonoid) were positively associated with the observed number of species. A prior study [7] identified a set of nasal microorganisms that offer protection against chronic rhinosinusitis, which were hence defined as protective nasal microorganisms in this study. The abundance of protective nasal microorganisms was positively associated with isoliquiritigenin (*p* = 0.0018, β = 0.002; a flavonoid) and serotonin (*p* = 0.0005, β = 0.62; an indole) but negatively associated with daily intake of rice/pasta/bread (*p* = 0.0091, β = −0.062).

As for oral microbiota, age starting kindergarten (*p* = 0.002, β = 0.48) was positively correlated with the Shannon index. The observed species showed significant associations with two flavonoids, tangeritin (*p* = 0.014, β = 2.29) and hesperidin (*p* = 0.005, β = 1.40). In line with a prior study [14] that identified protective and risky oral microorganisms for periodontitis, we defined these microorganisms as such in our study. The abundance of these risky oral microorganisms showed a positive association with early childhood exposure to smoking (*p* = 0.013, β = 0.005) and frequent consumption of fries (*p* = 0.019, β = 0.005). However, pyruvic acid abundance (*p* = 0.0173, β = −0.056) was negatively associated with risky oral microorganisms, while the total abundance of keto acids (*p* = 0.0147, β = 2.300) was positively correlated with oral protective microorganisms.

**Figure 4:**
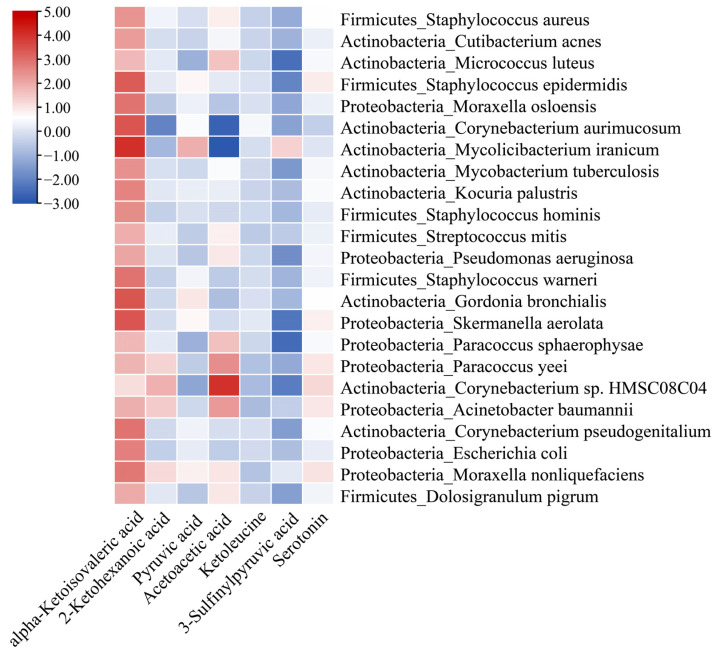
Co-occurrence probability of the top 25 indoor microbial species, keto acid derivatives, and serotonin.

Regarding indoor metabolites, flavonoids are predominantly plant-derived, whereas keto acids and indoles can be produced by indoor microorganisms. Therefore, neural network analysis [46] was employed to ascertain the co-occurrence probability of the top indoor microorganisms with serotonin and keto acids. The analysis revealed that certain indoor microorganisms, including *Staphylococcus epidermidis*, *Mycolicibacerium iranicum*, and *Corynebacterium* (Figure 4), co-occurred with these metabolites, indicating that these indoor microorganisms may potentially produce these metabolites.

### 3.6. Potential Microbial Transfer from Indoor Environment to Nasal/Oral Cavity

**Figure 5:**
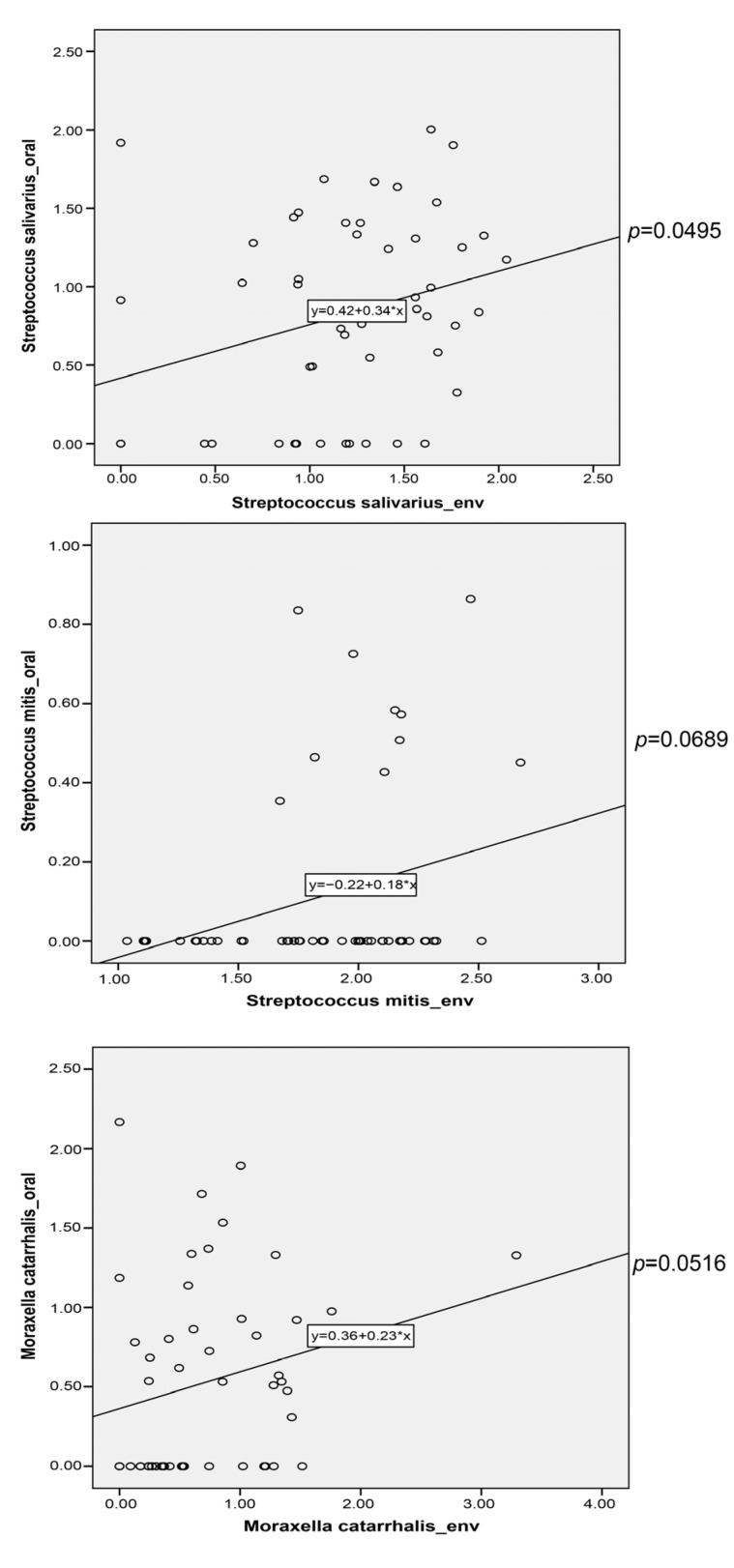
Spearman’s correlation of oral and indoor microbials in the indoor environment.

To investigate the potential transfer of microbes from the indoor environment to the nasal/oral cavity, we conducted a correlation analysis between major indoor and nasal/oral species. Three oral species, including *Moraxella catarrhalis* (rho = 0.2768, *p* = 0.0516), *Streptococcus mitis* (rho = 0.2564, *p* = 0.0689), and *Streptococcus salivarius* (rho = 0.2793, *p* = 0.0495), were positively correlated with the indoor species and could potentially be transmitted from an indoor environment to the oral cavity (Figure 5). We further analyze the VFs and ARGs in these species. In total, 8, 5, and 9 potential VFs were presented (RPKM > 0.5) in *M. catarrhalis* (*tufA*, *katA*, *clpE*, *hhuA*, *htpB*, *clpC*, *ccmF*, *carB*), *S. mitis* (*pavB*, *hasC*, *cbpA*, *pspA*), and *S. salivarius* (*pavB*, *hasC*, *psaA*, *fbp54*, *lap*, *cps4B*, *clpE*, *clpC*, *tufA*). These VFs were mainly involved in adherence, immune modulation, nutritional/metabolic factors, and stress survival. Similarly, 3, 3, and 2 potential ARGs were presented (RPKM > 0.5) in *M. catarrhalis* (*bro-1*, *icr-mc*, *vanY*), *S. mitis* (*patA*, *patB*, *vanY*), and *S. salivarius* (*vanY*, *vanT*). Interestingly, vancomycin resistance genes were widely distributed in these species, suggesting a potential acquisition of these ARGs in the oral cavity from the indoor environment.

## 4. Discussion

In the present study, we conducted a multi-omic analysis to explore the associations between the indoor microbiome/metabolites and the nasal/oral microbiota in children. We identified specific indoor flavonoids and mycotoxins, such as deoxynivalenol and nivalenol, that were significantly associated with microbial diversity and abundance in these cavities. Additionally, our data suggest that certain microorganisms may be transferred from the indoor environment to children’s oral cavities, potentially influencing virulence factors and antibiotic resistance profiles. These observations provide valuable insights into the complex interplay between the indoor environment and microbiota, opening new avenues for future research aimed at promoting human health.

### 4.1. Strengths and Limitations of the Study

This study has several notable strengths. Firstly, it pioneers the investigation of the relationship between indoor microbiome/metabolites and nasal/oral microbiota. Over 40 environmental and personal characteristics were collected, providing a comprehensive understanding of the effect of environmental factors on oral and nasal microbiota. Second, culture-independent multi-omics were utilized, including metagenomics, full-length 16S rRNA sequencing, and untargeted LC/MS. Multi-omic profiling has been widely conducted in intestinal gut studies [47] but is seldom reported in the indoor environment [32]. Therefore, our study can also provide resources for future analysis, such as comparing indoor microbiome/metabolite profiling with other environments. Thirdly, we used shotgun metagenomics to assess indoor functional genes, including VFs and ARGs.

Despite its contributions, this study has some limitations that warrant discussion. First, our study has a relatively small sample size of 56 healthy children, which was determined based on logistical constraints such as funding and available resources for in-depth multi-omics analyses. Although this sample size aligns with similar exploratory or pilot studies in this emerging research area, it does restrict the study’s statistical power for association analyses. Furthermore, the small sample size precluded us from applying stringent corrections for multiple testing, such as false discovery rate (FDR) control, as it would significantly reduce our ability to detect true associations. Therefore, our results should be interpreted as exploratory and will require validation in larger cohorts. Second, we used untargeted metabolomics for assessing indoor metabolites. While this approach allows for the characterization of a large number of metabolites, it yields only relative abundance, not absolute concentration. This restricts our ability to draw conclusions about the impacts of specific metabolite concentrations on the nasal and oral microbiota. In future studies, targeted metabolomics may enable more precise assessments of the impacts of specific indoor metabolites on the nasal/oral microbiota. Third, only one dust sample per child’s home was collected, raising questions about reproducibility over time and sensitivity to random variation. However, it should be noted that the vacuuming technique employed in this study is designed to collect large biomass samples. Prior research has shown that such samples are representative of long-term exposure and less susceptible to random variation compared to swab sampling [27]. Fourth, we did not conduct dental examinations to assess the oral health status of the participants. Thus, we cannot definitively conclude the precise oral health condition of our cohort. However, to ascertain the health status of the participants, the guardians of the children were interviewed and answered a comprehensive questionnaire. This method of data collection should lend some accuracy to our understanding of the participants’ health. Fifth, the source organisms for VFs and ARGs were identified using a bioinformatics approach, which may have inherent limitations that could lead to false positives. Similarly, the detection of species DNA in both oral and indoor environments does not necessarily indicate the viability or transferability of these organisms. Further studies investigating the survival and viability of these species in environmental conditions would be needed to confirm the actual transfer. Sixth, there is a methodological discrepancy in our approach, wherein we employed shotgun metagenomics for indoor samples and 16S rRNA sequencing for children’s nasal and oral samples. Shotgun metagenomics provides a comprehensive view of all the genetic material in the samples, including bacteria, viruses, fungi, and archaea, allowing for the identification of VFs, ARGs, and specific microbial genes. In contrast, 16S rRNA sequencing, while robust and cost-effective, specifically targets bacterial communities, potentially missing out on broader microbial diversity and specific gene-level information. This difference in methodologies could impact the comparability of the datasets and might have introduced biases in our analysis.

### 4.2. Indoor Metabolites and Nasal/Oral Microbiota

In this study, we found that indoor flavonoids (baicalein, eupatilin, isoliquiritigenin, tangeritin, and hesperidin) were associated with multiple nasal/oral microbial features, including the overall nasal microbial community variation, increased nasal/oral microbial alpha diversity, and an increased abundance of protective microorganisms in the oral cavity. These observations suggest a possible role for indoor flavonoid exposure in influencing nasal/oral health. Flavonoids are plant metabolites that possess several protective health effects, such as anti-bacterial, anti-oxidant, anti-inflammatory, anti-cancer, and anti-aging effects [48,49,50]. However, the mechanisms through which they increase nasal/oral diversity and the abundance of protective microbiota remain unclear. A possible mechanism is that flavonoids may suppress the biofilm formation and aggregation of pathogenic microorganisms. For instance, baicalein can protect oral health by inhibiting microbial biofilm formation, which prevents dental caries [51]. The suppression of pathogenic species may facilitate the growth of potentially beneficial microorganisms in the oral cavity.

Regarding the source of these flavonoids in the indoor environment, multiple possibilities exist. They could originate from outdoor plants, or they could be remnants from the consumption of flavonoid-rich foods and beverages within the household. However, it is important to clarify that the detected levels of dust might not directly indicate a health impact. Their presence might be due to higher consumption of flavonoid-rich foods in these environments, serving more as markers of certain dietary habits than direct influencers of health. Comparative analysis of flavonoid types and concentrations in outdoor plants and consumed foods would be essential for a more precise understanding of their origin and relevance.

In addition to flavonoids, we found that pyruvic acid (a keto acid derivative) and serotonin (an indole derivative) were associated with decreased risk microorganisms and increased protective microorganisms in the nasal/oral cavity. Pyruvic acid is a cellular metabolite produced at the end of glycolysis and has been widely used as a precursor for food, cosmetics, pharmaceuticals, and agricultural applications [52]. It has been shown to provide a wide range of beneficial health effects, including protection against oxidative stress and zinc toxicity [53,54]. However, their protective effects on the nasal/oral microbiota require further exploration. Serotonin is a monoamine neurotransmitter with multifaceted functions in modulating mood, cognition, learning, and memory. Approximately 90% of serotonin is produced in the human gut with the help of microorganisms, and therefore, it is one of the major signal transmitters in the gut–brain axis. A recent study in Malaysia reported that serotonin was present in high abundance in the dust of schools with low asthma prevalence [32], indicating that it may also play a role in respiratory health.

We found that nivalenol and deoxynivalenol in indoor dust were associated with the overall microbial composition in the nasal and oral cavities. Nivalenol and deoxynivalenol are mycotoxins that belong to the trichothecene group and are structurally similar. *Fusarium* species mainly produce these mycotoxins, and they are among the most widespread mycotoxins in food and feedstuffs [55]. Exposure to deoxynivalenol through food intake causes gastrointestinal inflammation and can lead to changes in the diversity, integrity, and composition of the gut microbiota, posing persistent threats to human and animal health [56]. However, the impact of nivalenol and deoxynivalenol on the nasal and oral microbiota has not been reported. In this study, we characterized these two mycotoxins in all samples, indicating their widespread presence in indoor dust. The resuspension of indoor dust and human inhalation may lead to nasal and oral exposure to these mycotoxins, which may further affect the nasal/oral microbiota.

### 4.3. Other Environmental and Personal Characteristics and Nasal/Oral Microbiota

In our study, we found a positive association between the presence of siblings and increased nasal microbial diversity. This finding is consistent with the Dutch Microbiome Project, which demonstrated that the number of cohabitants is the main factor in shaping the human microbiota [57]. Our results also showed that frequent intake of rice, pasta, bread, and French fries decreased the abundance of protective nasal microorganisms and increased the abundance of risky oral microorganisms. Although no previous studies have reported the association between food frequency and nasal/oral microbiota, our results are consistent with previous studies on gut microbiota. A high-glycemic and carbohydrate diet has been shown to decrease protective microorganisms and increase the risk of microorganisms in the gut microbiota [58,59]. Additionally, a high-fat diet, such as french fries, has been shown to promote the development of pro-inflammatory microbiota and alter the microbial composition of beneficial and pathogenic bacteria [60,61].

In our study, we did not find an association between air pollution and microbiota composition and diversity in the nasal and oral cavities, which is inconsistent with previous observations [17,18]. One potential explanation for this discrepancy is that we only sampled children in Shanghai for this study. The concentration of air pollutants in Shanghai was homogeneous across the entire city.

## 5. Conclusions

This study sheds light on the potential influence of the indoor microbiome and metabolites on the nasal and oral microbiota in children. The evidence points towards the crucial role of environmental factors, such as exposure to tobacco smoke and indoor flavonoids, in modulating the microbiota. Moreover, this study uncovers potential microbial transfer routes from indoor environments to the human body, warranting further research in this area. These findings underscore the critical need for a deeper understanding of our indoor microbial environments to promote health and prevent disease.

## Figures and Tables

**Table 1:** Associations between personal and environmental characteristics and the overall nasal and oral microbiota community of children (β-diversity). Metabolite abundances in the table are represented as intensity values derived from our untargeted metabolomics approach. The microbiota β-diversity was calculated using the Bray–Curtis distance indices. The associations were calculated using a 10,000 permutation bivariate Adonis/PERMANOVA analysis in R. Only associations with a *p*-value < 0.05 are shown in bold font.

		Nasal		Oral	
		R^2^	*p*-Value	R^2^	*p*-Value
**Personal characteristics**	Q1–Q3 percentile				
Age (year)	4–6	4.20	**0.008**	0.82	0.57
Breastfeeding duration (year)	0.5–1	0.82	0.49	1.02	0.38
Number of cohabitants	3–5	0.92	0.42	1.44	0.16
Age start kindergarten	3–4	1.45	0.21	1.23	0.25
Girl	59%	1.86	0.13	1.36	0.17
Preterm delivery	12%	0.38	0.82	0.58	0.75
Cesarean section	45%	0.33	0.84	0.95	0.46
Presence of siblings	51%	1.23	0.28	0.53	0.92
High parents income	14%	0.26	0.90	1.44	0.18
High education level for mother (graduate and postgraduate)	50%	1.00	0.38	0.51	0.83
High education level for father (graduate and postgraduate)	54%	2.82	**0.04**	0.51	0.89
**Food intake frequency**	Weekly/daily				
Juice and soda drink	86%/14%	2.10	0.09	0.98	0.41
Fries	90%/10%	0.90	0.43	0.49	0.81
Rice/pasta/bread	37%/63%	1.92	0.12	0.94	0.47
Fruits, vegetables	4%/96%	1.42	0.23	0.56	0.83
Eggs, milk, fish, meat, and sea foods	22%/78%	0.17	0.97	0.82	0.57
**Outdoor air pollution**					
SO_2_ (μg/m^3^)	4.75–10.78	1.40	0.23	0.57	0.78
NO2 (μg/m^3^)	29.35–43.10	1.66	0.17	1.20	0.25
CO (mg/m^3^)	0.59–0.76	1.15	0.32	0.67	0.74
O3 (μg/m^3^)	63.15–81.92	1.34	0.25	0.63	0.72
PM10 (μg/m^3^)	38.53–48.10	2.47	0.06	0.81	0.59
PM2.5 (μg/m^3^)	22.03–32.87	0.58	0.64	1.05	0.34
**Living environment characteristics**					
Adjacent to heavy traffic	41%	0.68	0.56	1.92	0.04
Adjacent to river/park/garden	22%	0.74	0.52	0.47	0.91
ETS mother—pregnancy	18%	4.63	**0.005**	1.74	0.09
ETS children—early childhood (<1 year)	14%	0.96	0.40	2.43	**0.03**
ETS children—previous 10 months	18%	1.87	0.13	1.17	0.30
Presence of pets/plants indoors—early childhood (<1 year old)	27%	1.55	0.19	1.29	0.22
Presence of pets/plants indoors—previous 10 months	59%	1.06	0.36	1.48	0.13
Visible mold/dampness—pregnancy	20%	1.41	0.22	1.39	0.18
Visible mold/dampness—early childhood (<1 year)	27%	1.01	0.37	0.77	0.65
Visible mold/dampness—previous 10 months	25%	1.06	0.35	0.80	0.61
Frequent room cleaning	38%	1.57	0.19	0.73	0.67
Building age (years)	10–40	0.40	0.79	0.42	0.96
**Abundance of potential pathogens indoor**	0.05–0.32	1.28	0.27	0.67	0.73
*Clostridium perfringens*	0.01%	0.92	0.42	0.35	0.86
*Salmonella enterica*	0.50%	0.47	0.73	0.88	0.48
*Listeria monocytogenes*	0.01%	1.14	0.31	1.15	0.28
*Toxoplasma gondii*	0.01%	0.52	0.69	0.74	0.56
*Mycobacterium tuberculosis*	1.47%	1.57	0.18	0.38	0.95
**Total abundance of VFs indoors (RPKM)**	2.3 × 10^3^–5.2 × 10^3^	0.92	0.42	0.61	0.81
**Total abundance of ARGs indoors (RPKM)**	2.4 × 10^3^–5.5 × 10^3^	0.91	0.42	0.67	0.75
**Abundance of flavonoids indoors**		3.91	**0.01**	1.08	0.35
Baicalein	0–2.86 × 10^10^	0.84	0.46	1.10	0.34
Daidzein	2.46 × 10^5^–1.18 × 10^8^	1.96	0.11	3.88	**0.03**
Tangeritin	1.92 × 10^6^–5.01 × 10^8^	1.58	0.18	0.69	0.60
Isoliquiritigenin	5.03 × 10^6^–2.85 × 10^8^	0.79	0.49	1.07	0.37
Apigenin	2.65 × 10^6^–5.31 × 10^8^	2.38	0.06	0.55	0.62
(2S)-Liquiritigenin	1.59 × 10^7^–2.22 × 10^9^	0.37	0.82	1.19	0.25
Hesperidin	2.31 × 10^6^–1.12 × 10^9^	2.45	0.07	1.78	0.09
Eupatilin	3.41 × 10^5^–1.32 × 10^8^	0.58	0.64	1.49	0.16
**Abundance of indoles indoors**		0.78	0.50	0.95	0.46
3-Methylindole	7.33 × 10^7^–1.64 × 10^9^	3.57	**0.01**	0.39	0.86
Serotonin	8.37 × 10^5^–6.52 × 10^8^	0.58	0.65	0.41	0.84
Indole	1.04 × 10^9^–5.03 × 10^9^	1.05	0.35	0.39	0.93
L-Tryptophan	1.67 × 10^9^–3.95 × 10^10^	0.73	0.54	2.14	**0.04**
Indole-3-carboxylic acid	7.50 × 10^5^–1.51 × 10^7^	1.04	0.36	0.28	0.99
**Abundance of keto acids indoors**		0.74	0.53	0.49	0.89
Pyruvic acid	3.51 × 10^7^–1.41 × 10^9^	0.75	0.52	0.99	0.37
Ketoleucine	1.43 × 10^7^–4.40 × 10^8^	2.74	**0.04**	1.17	0.26
2-Ketohexanoic acid	3.20 × 10^6^–2.95 × 10^8^	1.69	0.16	3.49	**0.04**
Acetoacetic acid	8.16 × 10^7^–1.28 × 10^10^	0.15	0.97	0.59	0.58
alpha-Ketoisovaleric acid	2.87 × 10^7^–9.61 × 10^8^	1.20	0.30	0.97	0.40
**Abundance of mycotoxins indoors**		1.83	0.14	1.41	0.17
Vomitoxin (deoxynivalenol)	7.30 × 10^4^–5.49 × 10^7^	3.26	**0.02**	1.34	0.19
Nivalenol	2.38 × 10^5^–1.81 × 10^7^	0.42	0.78	2.77	**0.03**
Tentoxin	8.60 × 10^7^–1.56 × 10^9^	2.14	0.09	1.78	0.10
Diacetoxyscirpenol	4.83 × 10^4^–1.75 × 10^7^	1.24	0.28	1.54	0.15

**Table 2:** Associations between personal information, environmental characteristics, α-diversity index, protective/risk nose, and oral microbial. The associations were calculated by linear regression adjusted for children’s age and gender. Only microorganisms with an abundance > 0.1% and metabolites with an abundance > 1 × 10^6^ (intensity) were included in the analysis. Associations with a *p*-value < 0.02 are presented in this table. Protective nasal microorganisms were defined based on a previous study in chronic rhinosinusitis [7] and included *Corynebacterium*, *Finegoldia*, *Anaerococcus*, *Peptoniphilus*, and *Staphylococcus*. Protective and risky oral microorganisms were defined based on a previous publication in periodontitis [14]. Risky oral microorganisms included *Treponema maltophilum*, *Treponema socranskii*, *Porphyromonas gingivalis*, *Tannerella forsythia*, *Treponema denticola*, *Parvimonas micra*, and *Selenomonas sputigena*, while protective oral microorganisms included *Rothia dentocariosab* and *Streptococcus sanguinis*.

	Coefficient	*p*-Value	95%CI	
**Nasal**				
**Shannon index**				
Presence of siblings	0.69	0.008	0.19	1.19
Baicalein (flavonoid)	0.72	0.004	0.24	1.20
**Observed number of species**				
Presence of siblings	29.9	0.01	7.49	52.3
Eupatilin (flavonoid)	0.68	0.004	0.23	1.12
**Protective microorganisms**				
Rice/pasta/bread	−0.06	0.009	−0.11	−0.02
Isoliquiritigenin (flavonoid)	0.002	0.002	0.001	0.003
Serotonin (indole)	0.62	0.0005	0.03	0.95
**Oral**				
**Shannon index**				
Age start kindergarten	0.48	0.002	0.18	0.77
Observed_OTU_env	−0.0009	0.011	−0.002	−0.0002
**Observed number of species**				
Tangeritin (flavonoid)	2.29	0.014	0.49	4.09
Hesperidin (flavonoid)	1.40	0.007	0.39	2.40
**Risk microorganisms**				
ETS children—early childhood (<1 year)	0.005	0.013	0.001	0.009
Fries	0.005	0.019	0.001	0.01
Pyruvic acid (keto acid)	−0.056	0.017	−0.10	−0.01
**Protective microorganisms**				
Total keto acid	2.30	0.015	0.47	4.10

## Data Availability

The sequencing data are in the Genome Sequence Archive https://ngdc.cncb.ac.cn/gsa/ (accessed on 26 September 2023) under the accession number PRJCA008482.
